# Brown Adipose Tissue Activation on ^18-^F-FDG-PET/CT Manifesting as Cachexia in a Patient With Pheochromocytoma

**DOI:** 10.1210/jcemcr/luae082

**Published:** 2024-05-14

**Authors:** Muhammad Najmi Md Nor, Kate Healy, John Feeney, Aoife Garrahy

**Affiliations:** Robert Graves Institute of Endocrinology, Tallaght University Hospital, D24 NR0A Dublin, Ireland; Department of Radiology, Tallaght University Hospital, D24 NR0A Dublin, Ireland; Department of Radiology, Tallaght University Hospital, D24 NR0A Dublin, Ireland; Robert Graves Institute of Endocrinology, Tallaght University Hospital, D24 NR0A Dublin, Ireland

**Keywords:** pheochromocytoma, PET/CT, brown adipose tissue, adrenal

## Image Legend

**Figure 1.**
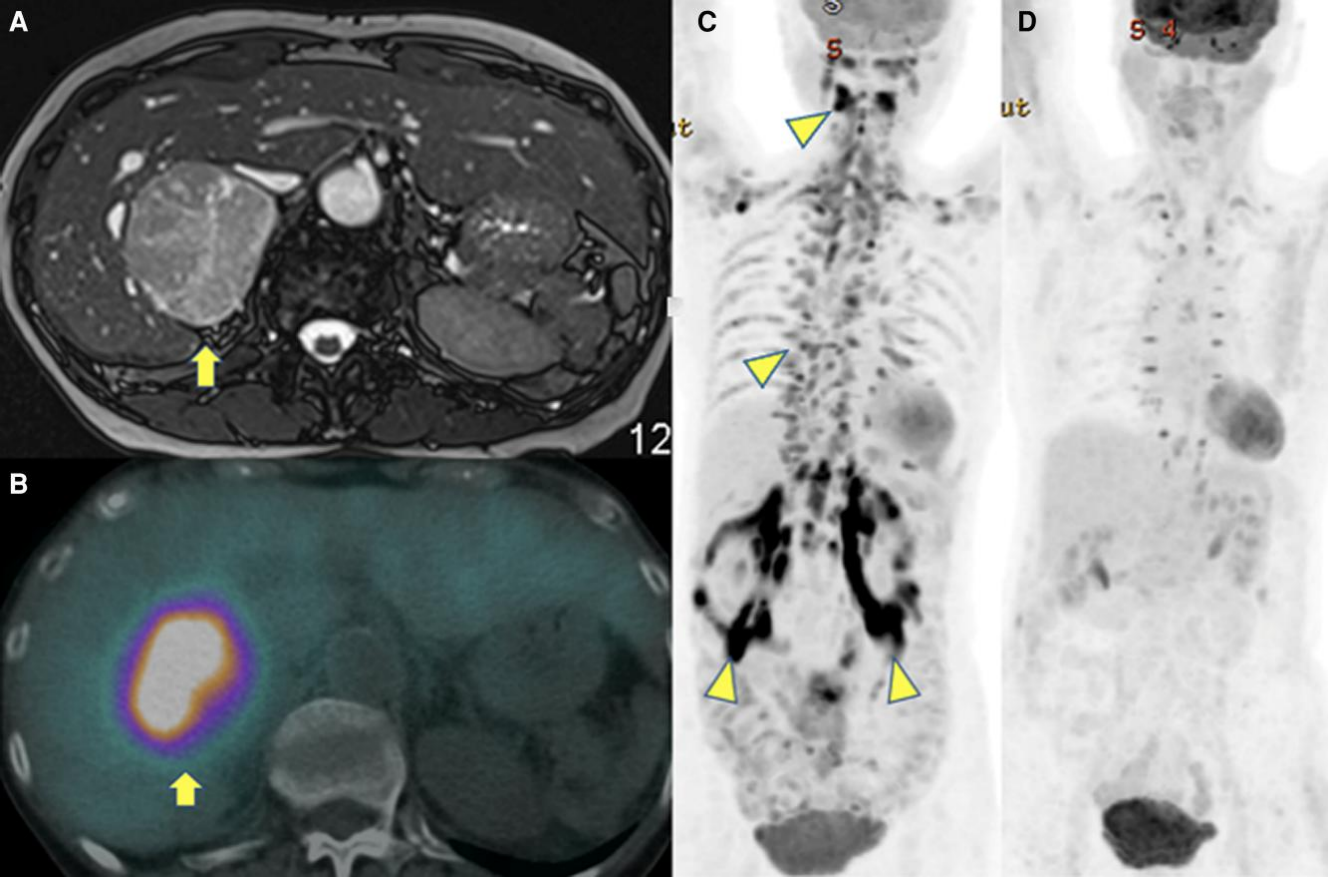
(A) MRI and (B) MIBG imaging demonstrating 7cm MIBG-avid right pheochromocytoma. (C) Pre-operative and (D) postoperative 18-F-FDG-PET/CT imaging demonstrating brown fat activation which resolved following resection of the tumour.

A 74-year-old woman was referred with a 7-cm right adrenal mass detected on computed tomography (CT) performed for investigation of weight loss. Medical history included hypertension requiring 3 antihypertensives. Biochemistry evaluation revealed elevated plasma normetanephrine, > 25 000 pmol/L (> 4578 pg/mL) (normal range: 0-1180 pmol/L; 0-216 pg/mL); metanephrine, > 16 000 pmol/L (> 3041 pg/mL) (normal range: 0-510 pmol/L; 0-97 pg/mL); and 3-methoxytyramine, 623 pmol/L (104 pg/mL) (normal range: 0-180 pmol/L; 0-30 pg/mL). Magnetic resonance imaging showed a 7-cm right adrenal mass (Fig. 1A). Iodine-123 metaiodobenzylguanidine (MIBG) single positron emission CT (SPECT/CT) showed an intensely MIBG-avid right adrenal mass (Fig. 1B). Fluorodeoxyglucose positron emission tomography CT (^18-^F-FDG-PET/CT) arranged in the initial weight loss evaluation showed uptake in the adrenal mass and within thoracic paravertebral and perinephric fat indicative of brown adipose tissue (BAT) activation (Fig. 1C). Elective open right adrenalectomy was performed, confirming a pheochromocytoma. Postoperatively, plasma metanephrines normalized. The ^18-^F-FDG-PET/CT showed normalization of paravertebral and perinephric FDG distribution and no FDG-avid disease (Fig. 1D). Norepinephrine-induced beta-adrenoceptor activation in patients with pheochromocytoma and paraganglioma can lead to BAT activation, which is associated with increased energy wasting and cachexia [1]. In this patient, this manifested as weight loss and BAT activation on ^18-^F-FDG-PET/CT, which were reversed following excision of the pheochromocytoma.

## Abbreviations

BAT: brown adipose tissue
CT: computed tomography
^18-^F-FDG-PET/CT: fluorodeoxyglucose positron emission tomography/computed tomography
MIBG: metaiodobenzylguanidine
